# Comparisons of short-term efficacy between individual and group cognitive behavioral therapy for primary insomnia

**DOI:** 10.1111/sbr.12019

**Published:** 2013-08-07

**Authors:** Wataru Yamadera, Miki Sato, Daisuke Harada, Masayuki Iwashita, Ryo Aoki, Keita Obuchi, Motohiro Ozone, Hiroshi Itoh, Kazuhiko Nakayama

**Affiliations:** Department of Psychiatry, Jikei University School of MedicineTokyo, Japan

**Keywords:** behavior and cognition, cognitive behavioral therapy for insomnia, insomnia, primary insomnia, psychology

## Abstract

The purpose of this study was to compare the efficacy of individual and group cognitive behavioral therapy for insomnia (CBT-I) in outpatients with primary insomnia diagnosed by DSM-IV-TR. The participants were 20 individually treated (I-CBT-I) and 25 treated in a group therapy format (three to five patients per group) (G-CBT-I), which showed no significant difference regarding demographic variables between groups. The same components of CBT-I stimulus control therapy, sleep restriction therapy, cognitive therapy, and sleep hygiene education were applied on both groups. The short-term outcome (4 weeks after treatment) was measured by sleep logs, actigraphy, the Pittsburgh Sleep Quality Index (PSQI), and the Dysfunctional Beliefs and Attitudes about Sleep Scale (DBAS), and was compared between I-CBT-I and G-CBT-I. The results indicated that CBT-I was effective in improving subjective and objective sleep parameters and subjective sleep evaluations for both individual and group treatment. However, I-CBT-I resulted in significantly better improvements over G-CBT-I, in (i) objective and subjective sleep onset latency time, (ii) objective sleep efficacy and moving time during sleeping, (iii) overall sleep quality and duration of actual sleep time in PSQI, (iv) consequences of insomnia, control and predictability of sleep, sleep requirement expectation, and sleep-promoting practices in DBAS. The present study suggested the superiority of I-CBT-I over G-CBT-I in clinical settings, and further evaluations are necessary.

## Introduction

Primary insomnia, as defined in the *Diagnostic and Statistical Manual of Mental Disorders*, 4th ed. text version (DSM-IV-TR),^1^ is the most common type of chronic insomnia and is almost the same concept as psychophysiological insomnia as defined in the *International Classification of Sleep Disorders* 2nd ed. (ICSD-2).^2^ Primary insomnia is characterized by morbid fear of insomnia, mental arousal, and heightened somatic tension in bed. Recently, it is been emphasized that cognitive behavioral therapy for insomnia (CBT-I) is effective for primary insomnia patients.^3^^–^6

The best tested and most commonly used method of delivering CBT-I had been via individualized treatment consisting of one-to-one sessions between a therapist and a single patient (I-CBT-I). As providing the I-CBT-I format is a time-consuming and cost-inefficient form of treatment delivery, the most common alternative delivery format is group therapy (G-CBT-I). However, no one established method of G-CBT-I has been used universally.^5^,^6^ Furthermore, whether I-CBT-I and G-CBT-I are equally efficacious is not clear. A previous meta-analysis suggested a modest superiority of I-CBT-T over G-CBT-I.^7^ On the other hand, a few clinical trials^8^,^9^ that provided direct comparisons of I-CBT-I and G-CBT-I within the same study mentioned no different outcomes between I-CBT-I and G-CBT-I. They concluded that G-CBT-I represented a cost-effective alternative to I-CBT-I for the management of insomnia. Although G-CBT-I is a popular approach, studies directly comparing the relative efficacy of individual and group formats are limited.^10^

The purpose of this study was to compare the short-term efficacy of I-CBT-I and G-CBT-I with the same treatment components and providers in clinically referred outpatients with primary insomnia in Japan. The primary outcomes were evaluated through subjective and objective sleep parameters, along with subjective sleep evaluations.

## Methods

### Study participants

The eligible subjects were sufferers of primary insomnia diagnosed by DSM-IV-TR, with chronic hypnotics use, attending Jikei University Hospital as outpatients, wishing to receive CBT-I. The participants for I-CBT-I consisted of 20 patients, and they participated in a study in 2004 to 2005.^11^ From 2009, the authors switched to G-CBT-I. The participants for G-CBT-I consisted of 25 patients divided over eight groups (three to five patients per group) in 2009 to 2011.^12^

The patients were excluded if they: (i) were 20 years of age or younger, (ii) met the DSM-IV-TR criteria for an axis I diagnosis of any psychiatric disorder and/or substance abuse, (iii) required psychotropic medication for psychiatric symptoms, or (iv) had possible sleep apnea syndrome (SAS) as judged from clinical interviews and daytime polysomnography (d-PSG).^13^ Each d-PSG was recorded from 14.00 hours to 04.00 hours. The respiratory tracings were evaluated for the presence of apnea (a 10-s or greater cessation of oronasal airflow) or hypopnea (a reduction in the amplitude of the thermistor signal by at least 50% for 10 s or longer, being followed by an electroencephalogram [EEG] arousal). To obtain the AHI (Apnea-Hypopnea Index), the number of apneas + hypopneas/total sleep time (TST, h) was calculated. The authors defined AHI ≥ 5 as the possible SAS, or (v) had symptoms suggestive of narcolepsy or restless legs syndrome as judged from clinical interviews.

The participants continued to take any medication already prescribed before enrollment, so as to avoid any effects of medication withdrawal during the treatment. The average daily dosage of hypnotics was calculated by dose equivalence of psychotropic drugs 2006-Version.^14^ A total of 53 patients (I-CBT-I: 24, G-CBT-I: 29) gave written informed consent to take part in the present study. During the treatment, however, eight patients (I-CBT-I: 4, G-CBT-I: 4) dropped out at their own request or at the recommendation of their attending physicians. Data from these patients were excluded from the statistical analysis.

### Treatment (Table 1)

**Table 1:** Outline of CBT-I protocol

	I-CBT-I	G-CBT-I
Treatment components	Stimulus control, sleep restriction cognitive restructuring, sleep hygiene education	
No. sessions	Three times	Two times (lecture and discussion, 60–90 min) plus one individual booster session (10 min)
	1st session: 60–90 min	
	2nd/3rd session: 15 min	
No. patients per group	Individually	3–5
Type of provider	Psychiatric sleep physician, MD	
Post-treatment evaluation	4 weeks after 1st session	4 weeks after 2nd session

The authors designed the CBT-I protocols with reference to the method described by Morin^5^ and Edinger.^6^ The four therapists (all men) conducted CBT-I, and all of them work as a clinical psychiatrist and a certified physician for a Japanese society involved in sleep research. The other authors supervised the contents of the CBT-I. The authors defined the 7 days prior to the first CBT-I session as the pre-treatment period to evaluate sleep-wake cycle of the patients for one week persistently. The post-treatment period was also defined as the first 7 days after the treatment or follow-up period (I-CBT-I: 4 weeks after the first session, G-CBT-I: 4 weeks after second session).

The I-CBT-I protocol was as follows.^11^ Just after the pre-treatment period, in the first 60–90-min session, after the introduction of CBT-I, the therapy was started for each individual patient by the same therapists. Thereafter, the patients underwent sessions of 15 min once every 2 weeks during the 4 weeks. A total of three sessions was given to each patient in this study. Four weeks after the first session, post-treatment evaluation was measured.

The G-CBT-I protocol was as follows.^12^ Just after the pre-treatment period, in the two-time group sessions (60–90 min, 3–5 patients per group, the interval was one week), patients participated in a lecture by a therapist and a group discussion of CBT-I. In addition, individual booster sessions (once, 10 min) were planned in the 4-week follow-up period, at one week or 2 weeks after the second session. Four weeks after the second session, post-treatment evaluation was measured.

The components of CBT-I were the same in both treatments. These consisted of stimulus control therapy,^5^,^6^ sleep restriction therapy,^5^,^6^ cognitive therapy,^5^,^6^,^11^,^12^ and sleep hygiene education.^5^–^6^

### Stimulus control therapy

Stimulus control attempts to break the association between the sleep environment and wakefulness by teaching the patients not to be engaged in activities that might disturb their sleep. The instructions the therapists gave were as follows: (i) go to bed only when becoming sleepy; (ii) do not use the bedroom for anything except sleep or sex; and (iii) get out of bed and go to another room whenever unable to fall asleep over a period of 30 min, and return to bed only when becoming sleepy again.

### Sleep restriction therapy

This treatment seeks to increase homeostatic sleep drive through partial sleep deprivation and thereby improve sleep ability. A bedtime and arising time schedule was prescribed in an attempt to improve sleep quality and decrease the time spent awake during the night. Time in bed was reduced in accordance with the total sleep time, as recorded in the sleep logs, and arising time was always fixed. The time the patient went to bed was adjusted on the basis of sleep efficiency. Though the authors were not absolutely strict in our administration of the sleep reduction therapy, combining with stimulus control therapy, the therapists emphasized the importance of spending time in bed only when sleepy.

### Cognitive therapy

As mentioned below, the therapists calculated the dissociation between the patients' subjective sleep evaluation from their sleep logs and their objective sleep data measured by an actigraph during the pre-CBT-I period. To facilitate better understanding by the patients, the therapists showed them the results of dissociation between the two parameters as an indicator of sleep state misperception. Subsequently, cognitive therapy was carried out to identify patient-specific incorrect cognition about sleep so that the therapists could correct any dysfunction in this regard.

### Sleep hygiene education

Sleep hygiene education included instruction about health practices and environmental factors that can be beneficial for maintaining sufficient sleep, and also details regarding homeostatic drive for sleep, circadian factors, and the effects of drugs and habits prior to sleep.

### Measurements

During the pre- and post-treatment periods, the authors conducted measurements including sleep logs, actigraphy, the Pittsburgh Sleep Quality Index (PSQI),^15^ and the Dysfunctional Beliefs and Attitudes about Sleep Scale (DBAS).^16^,^17^

### Sleep logs

During the pre- and post-treatment periods, patients were asked to complete sleep logs, just after getting up in the morning, for 7 days. Then, we averaged bedtime, rising time, sleep-onset time (SONT), sleep-offset time (SOFT), sleep onset latency time (SOL), total sleep time (TST), and total time in bed (TIB). In principle, bedtime and rising time on the sleep logs were recorded by each patient’s family members to increase the objectivity of the data.

### Actigraphy

During the pre- and post-treatment periods, patients were required to wear an actigraph (mini motionlogger actigraph; Ambulatory Monitoring, New York, NY, USA) on their non-dominant wrist at all times for 7 days. Based on the patient's rest/activity data recorded by actigraphy for 7 days, an estimation of their sleep was made using the algorithm devised by Cole *et al*.,^18^ which has a capability of more than 90% agreement with nocturnal PSG.^19^ From this result, the authors obtained the 7-day averaged data for objective SONT, SOFT, SOL, and the number of awakening episodes lasting more than 5 min (NOA), awakening time after sleep onset (WASO), TST, sleep efficiency (SE), and moving time during sleep (MT). SE was also calculated as the percentage of objective TST for each patient's actigraphy chart per TIB, recorded objectively on the sleep log by family members, as indicated above.

### Pittsburgh Sleep Quality Index

The authors assessed for sleep quality and quantity using the Japanese version of the Pittsburgh Sleep Quality Index (PSQI-J).^20^ PSQI-J consists of seven components, (i) overall sleep quality (SLPQUAL), (ii) sleep latency (LATEN), (iii) duration of actual sleep time (DURAT), (iv) sleep efficacy (HSE), (v) sleep disturbance (DISTB), (vi) medications necessary to sleep (MEDS), and (vii) day dysfunction due to sleepiness (DAYDYS). Each component was rated from 3 to 0, with global PSQI-J scores rating from 21 to 0.

### Dysfunctional Beliefs and Attitudes about Sleep Scale

Sleep-related cognition plays an important role in perpetuating insomnia, and it is reported that reduction of DBAS is correlated with improvement of sleep parameters. Therefore in the present study, the authors used the Japanese version of the DBAS (DBAS-J)^21^ to grasp the patients’ faulty cognition about sleep. The DBAS is a self-recorded questionnaire developed by Morin *et al*.^17^ It consists of a 28-item scale that extracts various beliefs and attitudes about sleep, focusing on the following five themes: (i) consequences of insomnia, (ii) control and predictability of sleep, (iii) sleep requirement expectations, (iv) causal attributes of insomnia, and (v) sleep-promoting practices. A higher score indicates a more dysfunctional belief. The average scores for the five themes were compared between the pre- and post-treatment situations.

### Statistical analysis

Data were analyzed using Stat View-J5.0 for Windows (SAS Institute, Tokyo, Japan). Each parameter for demographic data was compared between the two groups, I-CBT-I and G-CBT-I, using the unpaired *t*-test or χ^2^ test. Analysis of variance (anova) with repeated measures was used to determine variances over time (pre-treatment and post-treatment), between the two groups (I-CBT-I and G-CBT-I), along with the interaction of the group over time. *P*-values of group difference were calculated using pre-treatment data. Statistical significance was determined at *P* < 0.05.

### Approval of the study

The study protocol and therapy regimen were approved by the Jikei University School of Medicine Ethics Committee. Written informed consent to participate in the study was obtained from all the participants after they were given an explanation of the study and its potential risks. All of the procedures were carried out in accordance with Good Clinical Practice, the Helsinki Declaration, and related laws.

## Results

Table 2 shows comparisons of demographic and clinical variables; no significant differences were seen between the patients of I-CBT-I and G-CBT-I regarding age, sex, duration of insomnia, daily dosage of hypnotics, pre-treatment global PSQI-J scores, and the numbers of dropout patients.

**Table 2:** Comparison of demographic variables for the patients between I-CBT-I and G-CBT-I

	I-CBT-I (*n* = 20)	G-CBT-I (*n* = 25)
Age (years, [range])	56.9 ± 12.6 [27–76]	61.7 ± 11.3 [35–81]
Sex (M:F, [%male])	6:14 [30.0]	14:11 [56.0]
Duration of insomnia (years, [range])	8.9 ± 6.2 [1–22]	8.0 ± 6.4 [0.5–21]
Dairy dosage of hypnotics (flunitrazepam1 mg = 1, [range])	1.6 ± 1.2 [0–4.0]	1.9 ± 1.1 [0.33-4.0]
Pre-treatment global PSQI-J scores [range]	12.7 ± 3.0 [6–18]	12.2 ± 2.4 [9–17]
No. drop-out patients (%)	4/24 (16.7)	4/29 (13.8)

Mean ± SD or N unpaired *t*-test, χ^2^ test: N.S. Duration of insomnia: the subjectively reported period from initial appearance of insomnia to the time of receiving CBT-I. PSQI-J: the Japanese version of the Pittsburgh Sleep Quality Index.

**Figure 1:**
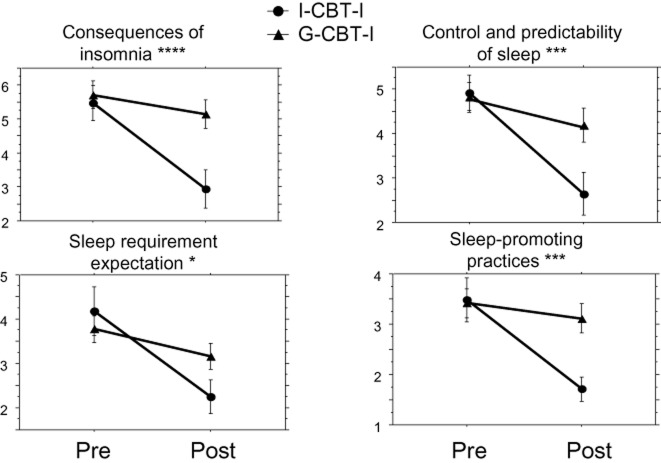
Comparison of changes in sleep-related cognition (DBAS-J) between I-CBT-I and G-CBT-I. DBAS-J: Dysfunctional Beliefs and Attitudes about Sleep Scale, Japanese version. Mean ± SE, Analysis of variance (anova) with repeated measures, G × T: interaction, *P < 0.05, ***P < 0.005, ****P < 0.001.

Table 3 through to Table 6 shows the results from sleep logs (Table 3), actigraphy (Table 4), PSQI-J (Table 5), and DBAS-J (Table 6). The results of anova with repeated measures were as follows. There was a significant group effect for NOA (*P* = 0.010) as measured by actigraphy and for DISTB (*P* = 0.032) as measured by PSQI-J. There was a significant time effect for bed time (*P* = 0.003), rising time (*P* = 0.001), SOL (*P* = 0.001), and TST (*P* = 0.009), as measured by sleep logs, for SOL (*P* = 0.001), WASO (*P* = 0.001), TIB (*P* = 0.001), SE (*P* = 0.001), and MT (*P* = 0.006), as measured by actigraphy, for SLPQUAL (*P* = 0.001), LATEN (*P* = 0.001), DURAT (*P* = 0.001), HSE (*P* = 0.034), global PSQI-J (*P* = 0.001) as measured by PSQI-J and for consequences of insomnia (*P* = 0.001), control and predictability of sleep (*P* = 0.001), sleep requirement expectations (*P* = 0.001), and sleep-promoting practices (*P* = 0.001) as measured by DBAS-J. Further, there was a significant group × time interaction for SOL (*P* = 0.004) as measured by sleep logs, for SOL (*P* = 0.001), SE (*P* = 0.017), and MT (*P* = 0.046) as measured by actigraphy, for SLPQUAL (*P* = 0.046) and DURAT (*P* = 0.023) as measured by PSQI-J, and for consequences of insomnia (*P* = 0.001), control and predictability of sleep (*P* = 0.001), sleep requirement expectations (*P* = 0.040), and sleep-promoting practices (*P* = 0.004) as measured by DBAS-J. Figure 1 shows a comparison of changes in the themes of DBAS-J between I-CBT-I and G-CBT-I.

**Table 3:** Comparison of changes in sleep logs between I-CBT-I and G-CBT-I

	Pre-treatment		Post-treatment		*P*-value		
	I-CBT-I	G-CBT-I	I-CBT-I	G-CBT-I	G	T	G × T
Bedtime (h)	23.1 ± 0.3	23.3 ± 0.3	23.7 ± 0.2	23.6 ± 0.2	0.917	0.003*	0.209
Rising time (h)	7.2 ± 0.2	7.1 ± 0.2	6.8 ± 0.2	6.8 ± 0.2	0.836	0.001*	0.738
SONT (h)	24.3 ± 0.3	24.1 ± 0.3	23.8 ± 0.3	24.1 ± 0.2	0.819	0.236	0.261
SOFT (h)	5.9 ± 0.3	5.7 ± 0.2	6.1 ± 0.3	6.0 ± 0.2	0.695	0.069	0.897
SOL (min)	69.3 ± 8.5	46.9 ± 6.0	26.3 ± 3.4	31.5 ± 2.7	0.181	0.001*	0.004*
TST (min)	328.6 ± 17.4	324.8 ± 12.7	351.9 ± 10.3	348.2 ± 7.7	0.807	0.009*	0.999

Mean ± SE, I-CBT-I: individual CBT-I (*n* = 20), G-CBT-I, group CBT-I (*n* = 25). Analysis of variance (anova) with repeated measures, G, groups (individual vs group), T, time (Pre-treatment vs Post-treatment), G × T, interaction, *P*-values of Group difference were calculated using pre-treatment data.

* < 0.05. SOFT, sleep offset time; SOL, sleep onset latency; SONT, sleep onset time; TST, total sleep time.

**Table 4:** Comparison of changes in actigraphy between I-CBT-I and G-CBT-I

	Pre-treatment		Post-treatment		*P* value		
	I-CBT-I	G-CBT-I	I-CBT-I	G-CBT-I	G	T	G × T
SONT (h)	23.6 ± 0.3	23.7 ± 0.3	23.8 ± 0.2	24.0 ± 0.2	0.766	0.105	0.916
SOFT (h)	6.8 ± 0.3	6.6 ± 0.2	6.7 ± 0.2	6.5 ± 0.2	0.497	0.290	0.945
SOL (min)	30.4 ± 6.2	20.0 ± 2.2	7.2 ± 1.0	20.1 ± 2.8	0.743	0.001*	0.001*
NOA (times)	3.1 ± 0.7	1.5 ± 0.2	2.9 ± 0.7	1.4 ± 0.1	0.010*	0.487	0.991
WASO (min)	24.9 ± 4.1	15.9 ± 2.8	15.8 ± 2.5	12.5 ± 2.1	0.109	0.001*	0.098
TIB (min)	475.5 ± 18.6	458.4 ± 12.6	425.6 ± 10.3	428.3 ± 8.5	0.636	0.001*	0.309
TST (min)	397.0 ± 10.8	388.1 ± 11.5	391.4 ± 9.3	376.0 ± 9.6	0.377	0.146	0.589
SE (%)	84.4 ± 1.9	85.5 ± 1.7	92.1 ± 0.8	88.5 ± 1.4	0.522	0.001*	0.017*
MT (counts/min)	10.4 ± 1.0	8.2 ± 0.9	8.4 ± 0.8	7.9 ± 0.8	0.254	0.006*	0.046*

Mean ± SE, I-CBT-I: individual CBT-I (*n* = 20), G-CBT-I: group CBT-I (*n* = 25). Analysis of variance (anova) with repeated measures, G: groups, T: time, G × T, interaction, *P*-values of Group difference were calculated using pre-treatment data.

* *P* < 0.05. MT, moving time during sleeping; NOA, numbers of awakening episodes lasting more than 5 min; SE, sleep efficiency; TIB, total time in bed; WASO, awakening time after sleep onset.

**Table 5:** Comparison of changes in PSQI-J between I-CBT-I and G-CBT-I

	Pre-treatment		Post-treatment		*P*-value		
	I-CBT-I	G-CBT-I	I-CBT-I	G-CBT-I	G	T	G × T
SLPQUAL (C1)	2.3 ± 0.1	2.1 ± 0.1	1.2 ± 0.1	1.5 ± 0.1	0.443	0.001*	0.046*
LATEN (C2)	2.4 ± 0.2	2.0 ± 0.2	1.6 ± 0.2	1.4 ± 0.2	0.242	0.001*	0.310
DURAT (C3)	2.3 ± 0.2	1.8 ± 0.2	1.4 ± 0.2	1.6 ± 0.1	0.430	0.001*	0.023*
HSE (C4)	1.6 ± 0.3	1.4 ± 0.2	0.9 ± 0.2	1.0 ± 0.2	0.920	0.034*	0.461
DISTB (C5)	1.0 ± 0.1	1.2 ± 0.1	1.0 ± 0.1	1.2 ± 0.1	0.032*	0.999	0.999
MEDS (C6)	2.5 ± 0.2	2.8 ± 0.1	2.4 ± 0.3	2.8 ± 0.1	0.144	0.352	0.589
DAYDYS (C7)	0.8 ± 0.2	0.8 ± 0.1	0.5 ± 0.1	0.8 ± 0.1	0.213	0.150	0.295
Global PSQI-J	12.7 ± 0.7	12.2 ± 0.5	8.9 ± 0.6	10.1 ± 0.7	0.615	0.001*	0.066

PSQI-J: the Japanese version of Pittsburgh Sleep Quality Index, I-CBT-I: individual CBT-I (*n* = 20), G-CBT-I: group CBT-I (*n* = 25), mean ± SE, Analysis of variance (anova) with repeated measures, G: groups, T: time, G × T: interaction, *P*-values of Group difference were calculated using pre-treatment data.

* *P* < 0.05. dayDYS, day dysfunction due to sleepiness; DISTB, sleep disturbance; DURAT, duration of actual sleep time; HSE, sleep efficiency; LATEN, sleep latency; MEDS, need medications to sleep; SLPQUAL, overall sleep quality.

**Table 6:** Comparison of changes in DBAS-J between I-CBT-I and G-CBT-I

	Pre-treatment		Post-treatment		*P*-value		
	I-CBT-I	G-CBT-I	I-CBT-I	G-CBT-I	G	T	G × T
Consequences of insomnia	5.5 ± 0.5	5.7 ± 0.4	3.0 ± 0.6	5.1 ± 0.4	0.053	0.001*	0.001*
Control and predictability of sleep	4.9 ± 0.4	4.8 ± 0.3	2.7 ± 0.5	4.2 ± 0.4	0.163	0.001*	0.001*
Sleep requirement expectation	4.2 ± 0.6	3.8 ± 0.3	2.3 ± 0.4	3.2 ± 0.3	0.567	0.001*	0.040*
Causal attributions of insomnia	3.4 ± 0.5	3.7 ± 0.5	2.2 ± 0.5	3.5 ± 0.4	0.153	0.074	0.179
Sleep-promoting practices	3.5 ± 0.4	3.4 ± 0.3	1.7 ± 0.2	3.1 ± 0.3	0.091	0.001*	0.004*

DBAS-J, Dysfunctional Beliefs and Attitudes about Sleep scale Japanese version. I-CBT-I, individual CBT-I (*n* = 20), G-CBT-I: group CBT-I (*n* = 25), mean ± SE. Analysis of variance (anova) with repeated measures. G, groups; T, time; G × T, interaction; *P*-values of Group difference were calculated using pre-treatment data.

* *P* < 0.05.

## Discussion

Controlled trials have established the efficacy of CBT-I for primary insomnia.^5^,^6^ However, the relative efficacy of individual versus group treatment formats in real-world settings is not well established. The present study compared the short-term efficacy in a clinical setting between I-CBT-I and G-CBT-I for primary insomnia outpatients undergoing the same treatment components and providers. This trial represents the first attempt to compare different formats of CBT-I using actigraphy as the objective sleep measurement. As there is reportedly an underestimation of objective sleep evaluation and dissociations between subjective and objective evaluation of sleep in primary insomnia,^22^ it is important to evaluate the therapeutic changes in objective measurements.

The findings in the present study complemented previous studies^3^,^4^,^7^ showing that CBT-I was effective treatment for primary insomnia. The comparison of pre-treatment to post-treatment, as the short-term outcome, showed that CBT-I produced significant changes in many parameters. In the post-treatment measurements, subjective bedtime was delayed, subjective rising time was advanced, subjective TST increased, subjective and objective SOL shortened, objective TIB, WASO, and MT decreased, and objective SE increased. All of these suggested the improvement of nocturnal sleep after the treatment. At the same time, subjective evaluations of sleep quality and quantity improved, and the patients’ faulty cognition about sleep was corrected after the treatment.

In the present study, different outcomes between I-CBT-I and G-CBT-I were shown. In regard to objective and subjective sleep onset latency time, objective sleep efficacy and moving time during sleeping, overall sleep quality and duration of actual sleep time in PSQI, the consequences of insomnia, control, and predictability of sleep, sleep requirement expectation, and sleep-promoting practices in DBAS, I-CBT-I resulted in larger improvements compared with G-CBT-I. Although these results were contrasted with the previous studies^8^,^9^,^23^ the present study suggested a slight superiority of I-CBT-I over G-CBT-I in the improvements of not only subjective but also objective sleep measurements. Furthermore, the superiority was remarkable in the correction of dysfunctional beliefs and attitudes about sleep. One reported advantage of I-CBT-I is its fit within traditional medical and mental health outpatient settings. I-CBT-I also allows maximum flexibility in tailoring treatment to best address each individual patient’s problematic sleep-related cognition and behavior.^6^ On the other hand, G-CBT-I may afford patients less individualized attention.^6^ A possible explanation will be able to propose that patients who are more severely insomniac or socially anxious may generally find it harder to engage in group treatment, and therapists in a group setting may have less opportunity to address specific patient needs. Most previously described G-CBT-I protocols were typically provided in four to eight treatment sessions to a group of patients.^6^ Another explanation will be able to propose that numbers and hours spent in the sessions in this study may be insufficient. It will be important for future research to determine if individual and group CBT-I have a similar or different relationship to the maintenance of efficacy in routine clinical settings.

Concerning CBT for depression, a clinical study that investigated outcome, costs, and patient engagement for group and individual CBT for depression mentioned that no significant differences were found in depressive symptoms between group and individual CBT at post-treatment and follow-up, and concluded that there were no differences between group differences in attrition or satisfaction.^24^ Another recent study that evaluated the effectiveness of group CBT compared to individual CBT for depressed outpatients mentioned that individual CBT was associated with larger effect sizes and significantly higher rates of recovery compared with group CBT.^25^ Regardless, systematic cost-effectiveness and cost-benefit comparisons between individual and group CBT for primary insomnia or depression have not yet been conducted. From a cost-effectiveness perspective, however, there are certainly advantages to implementing treatment in a group format.^5^,^6^ Future research should seek to replicate these findings under similar and controlled conditions, and to establish the comparative cost-effectiveness of each format of treatment.

The present study has several limitations. The first limitations of the present study lie in the fact that there was no randomization for I-CBT-I and G-CBT-I. The periods of implements were also different for a few years. Not all of the present results can be considered to indicate the efficacy of CBT-I. All of the participants wished to receive CBT-I and therefore might have been very motivated. The second limitation lies in the fact that there was no control group. In the absence of a control group, it is impossible to rule out improvements over time. The third limitation lies in the fact that the authors could observe and compare only the short-term outcome. It is important to keep in mind and follow up on the fact that primary insomnia patients who benefit from short-term evaluation might remain vulnerable to recurrent insomnia episodes in the long term. In the present study, however, at 6 months after the treatment, sleep improvements and drug tapering achieved with G-CBT-I patients were well sustained.^12^ Although these limitations should be further discussed, in conclusion, the findings of the present study support the view that in a more clinically representative setting, I-CBT-I is a superior treatment format for primary insomnia compared to G-CBT-I.
